# Educational Attainment and Ischemic Stroke: A Mendelian Randomization Study

**DOI:** 10.3389/fgene.2021.794820

**Published:** 2022-02-11

**Authors:** Luyan Gao, Kun Wang, Qing-Bin Ni, Hongguang Fan, Lan Zhao, Lei Huang, Mingfeng Yang, Huanming Li

**Affiliations:** ^1^ Department of Neurology, Tianjin Fourth Central Hospital, The Fourth Central Hospital Affilicated to Nankai University, The Fourth Central Clinical College of Tianjin Medical University, Tianjin, China; ^2^ Taishan Academy of Medical Sciences, Taian City Central Hospital, Taian, China; ^3^ Second Affiliated Hospital, Brain Science Institute, Key Laboratory of Cerebral Microcirculation in Universities of Shandong, Shandong First Medical University and Shandong Academy of Medical Sciences, Taian, China; ^4^ Department of Cardiovascular, Tianjin Fourth Central Hospital, The Fourth Central Hospital Affilicated to Nankai University, The Fourth Central Clinical College of Tianjin Medical University, Tianjin, China

**Keywords:** stroke, educational attainment, Mendelian randomization, genome-wide association studies, ischaemic stroke

## Abstract

Observational studies have evaluated the potential association of socioeconomic factors such as higher education with the risk of stroke but reported controversial findings. The objective of our study was to evaluate the potential causal association between higher education and the risk of stroke. Here, we performed a Mendelian randomization analysis to evaluate the potential association of educational attainment with ischemic stroke (IS) using large-scale GWAS datasets from the Social Science Genetic Association Consortium (SSGAC, 293,723 individuals), UK Biobank (111,349 individuals), and METASTROKE consortium (74,393 individuals). We selected three Mendelian randomization methods including inverse-variance-weighted meta-analysis (IVW), weighted median regression, and MR–Egger regression. IVW showed that each additional 3.6-year increase in years of schooling was significantly associated with a reduced IS risk (OR = 0.54, 95% CI: 0.41–0.71, and *p* = 1.16 × 10^–5^). Importantly, the estimates from weighted median (OR = 0.49, 95% CI: 0.33–0.73, and *p* = 1.00 × 10^–3^) and MR–Egger estimate (OR = 0.18, 95% CI: 0.06–0.60, and *p* = 5.00 × 10^–3^) were consistent with the IVW estimate in terms of direction and magnitude. In summary, we provide genetic evidence that high education could reduce IS risk.

## Introduction

Stroke is one of the leading causes of serious long-term disability in the world and is the fifth leading cause of death in the United States (Mozaffarian et al., 2016a; Mozaffarian et al., 2016b). Every year, there are more than 795,000 people having a stroke and more than 130,000 deaths from stroke, and the estimated stroke cost is $33 billion in the United States (Mozaffarian et al., 2016b). In recent years, there has been an increased interest for observational studies exploring the impact of socioeconomic factors such as higher education on stroke risk. In fact, a number of studies have reported that high education could reduce the risk of stroke (Nordahl et al., 2014; Ferrario et al., 2017; Kubota et al., 2017; Mchutchison et al., 2017). However, there are still some inconsistent findings. In 2002, Chang et al. found that stroke risk was reduced among less educated women in Africa, compared to highly educated women (Chang et al., 2002). It is well known that persons with cognitive impairment are at a higher risk of stroke (Sajjad et al., 2015). In 2015, Sajjad et al. conducted an observational study of 9,152 participants from the Rotterdam (Sajjad et al., 2015). They identified that education could modify the association between subjective memory complaints and risk of stroke (Sajjad et al., 2015). Higher education is significantly associated with a higher risk of stroke (hazard ratio = 1.39; 95% CI: 1.07–1.81) (Sajjad et al., 2015).

In recent years, large-scale genome-wide association studies (GWAS) promptly identified some common genetic variants and provided insight into the genetics of educational attainment (Okbay et al., 2016) and stroke (Malik et al., 2016). The existing large-scale GWAS datasets provide strong support for investigating the potential causal association of educational attainment with stroke risk by a Mendelian randomization analysis (Mokry et al., 2015; Nelson et al., 2015; Ference et al., 2017; Larsson et al., 2017a; Manousaki et al., 2017; Tillmann et al., 2017). This method could avoid some limitations of observational studies and is widely used to determine the causal inferences (Mokry et al., 2015; Ference et al., 2017; Larsson et al., 2017a; Manousaki et al., 2017; Tillmann et al., 2017; Wang et al., 2020).

It is reported that about 87% of all strokes are ischemic stroke (IS), in which blood flow to the brain is blocked (Mozaffarian et al., 2016a; Mozaffarian et al., 2016b). Intracerebral hemorrhage is the second most common cause of stroke (about 15%–30% of strokes) (An et al., 2017). Here, we performed a Mendelian randomization (MR) study to investigate the association of increased educational attainment with IS risk using the genetic variants from the large-scale educational attainment GWAS dataset (*N* = 405,072 individuals of European descent) and the large-scale IS GWAS dataset (*N* = 29,633, including 10,307 IS cases and 19,326 controls of European descent).

## Materials and Methods

### Study Mesign

MR is based on three principal assumptions (Emdin et al., 2017; Larsson et al., 2017a). First, the genetic variants selected to be instrumental variables should be associated with the exposure (educational attainment) (Emdin et al., 2017; Larsson et al., 2017a). Second, the genetic variants should not be associated with confounders (assumption 2) (Emdin et al., 2017; Larsson et al., 2017a). Third, genetic variants should affect the risk of the outcome (IS) only through the exposure (educational attainment) (assumption 3) (Emdin et al., 2017; Larsson et al., 2017a). Recent studies have provided the more detailed information about the three principal assumptions (Liu et al., 2018; Liu et al., 2019; Zhang et al., 2020; Liu et al., 2021a; Liu et al., 2021b; Sun et al., 2021). This study is based on the publicly available, large-scale GWAS summary datasets. All participants gave informed consent in all these corresponding original studies. All relevant data are within the paper and the Supplementary Tables S1. The authors confirm that all data underlying the findings are either fully available without restriction through consortia websites or may be made available from consortia upon request.

### Educational Attainment GWAS Dataset

We selected a large-scale GWAS dataset of educational attainment in individuals of European descent whose educational attainment was assessed at or above age 30 (Okbay et al., 2016). The examined phenotype is a continuous variable measuring the number of years of schooling completed (EduYears) (Okbay et al., 2016). This GWAS dataset consisted of 293,723 individuals in the discovery stage [Social Science Genetic Association Consortium (SSGAC), EduYears mean = 14.3, standard deviation (SD) = 3.6] and 111,349 individuals in the independent replication stage (UK Biobank, EduYears mean = 13.7, SD = 5.1) (a total of 405,072 individuals of European descent) (Okbay et al., 2016). In brief, the discovery stage GWAS from SSGAC was performed at the cohort level in individuals of European descent (Okbay et al., 2016). The replication stage GWAS from UK Biobank was conducted using conventionally population-based unrelated individuals with “White British” ancestry in the United Kingdom (Okbay et al., 2016). The meta-analysis of the discovery and replication stages of GWAS identified 162 independent genetic variants with the genome-wide significance (*p* < 5.00 × 10^–8^) (Okbay et al., 2016). Here, we selected these 162 independent genetic variants as the potential instrumental variables, as provided in Table 1 and Supplementary Table S1, which could explain 1.6%–1.8% of the variance in education (Tillmann et al., 2017). Meanwhile, Li and others also selected these 162 independent genetic variants in their MR analysis to evaluate the causal association between educational attainment and asthma (Li et al., 2021).

**TABLE 1:** 162 independent genetic variants as the potential instrumental variables.

SNP	Effect allele	Non-effect allele	Effect allele frequency	Effect size	Standard error	*p*-value
rs11130222	A	T	0.59	0.025	0.0023	3.68E-28
rs13090388	T	C	0.31	0.026	0.0024	2.58E-26
rs7029201	A	G	0.41	0.025	0.0023	7.16E-27
rs9401593	A	C	0.52	−0.024	0.0022	3.83E-28
rs12987662	A	C	0.39	0.025	0.0023	8.52E-28
rs8002014	A	G	0.27	−0.024	0.0025	3.80E-21
rs34305371	A	G	0.1	0.036	0.0039	1.52E-20
rs10773002	A	T	0.25	0.022	0.0026	7.74E-18
rs6882046	A	G	0.74	−0.019	0.0025	8.12E-14
rs17824247	T	C	0.59	−0.016	0.0023	2.41E-12
rs61160187	A	G	0.61	−0.017	0.0023	2.71E-14
rs11588857	A	G	0.21	0.02	0.0027	3.27E-13
rs2456973	A	C	0.67	−0.019	0.0024	5.83E-16
rs10786662	C	G	0.55	−0.017	0.0022	4.63E-14
rs4863692	T	G	0.32	0.017	0.0024	4.61E-12
rs10223052	A	G	0.36	0.016	0.0023	3.56E-12
rs11998763	A	G	0.54	0.017	0.0022	4.61E-14
rs9964724	T	C	0.68	0.018	0.0024	2.39E-14
rs6839705	A	C	0.36	0.015	0.0023	1.19E-10
rs7964899	A	G	0.44	0.016	0.0022	4.37E-13
rs12410444	A	G	0.7	−0.017	0.0024	6.01E-13
rs112634398	A	G	0.95	0.038	0.0055	2.74E-12
rs1106761	A	G	0.38	−0.016	0.0023	1.37E-11
rs3172494	T	G	0.12	0.023	0.0036	8.98E-11
rs58694847	C	G	0.26	−0.018	0.0025	4.98E-12
rs1008078	T	C	0.4	−0.017	0.0023	3.10E-14
rs34344888	A	G	0.39	−0.016	0.0023	8.87E-13
rs1378214	T	C	0.37	−0.015	0.0023	1.85E-11
rs16845580	T	C	0.63	0.016	0.0023	4.14E-12
rs12900061	A	G	0.18	0.019	0.0029	5.04E-11
rs35771425	T	C	0.79	0.018	0.0027	2.71E-11
rs7776010	T	C	0.82	−0.021	0.003	2.61E-12
rs1338554	A	G	0.5	0.015	0.0022	1.52E-11
rs356992	C	G	0.3	0.017	0.0024	4.03E-12
rs7593947	A	T	0.51	0.015	0.0022	2.39E-11
rs1912528	T	C	0.36	0.014	0.0023	1.53E-09
rs2992632	A	T	0.72	0.016	0.0025	3.25E-11
rs4741351	A	G	0.3	−0.015	0.0024	2.98E-10
rs6715849	A	G	0.44	−0.015	0.0022	1.65E-11
rs660001	A	G	0.21	−0.018	0.0027	1.34E-10
rs320700	A	G	0.65	0.014	0.0023	3.91E-09
rs113474297	T	C	0.13	−0.021	0.0034	8.34E-10
rs28420834	A	G	0.45	−0.014	0.0023	2.67E-10
rs56231335	T	C	0.67	−0.017	0.0024	7.17E-13
rs62263923	A	G	0.64	−0.017	0.0023	1.11E-13
rs12076635	C	G	0.79	0.018	0.0027	3.11E-11
rs9556958	T	C	0.53	−0.015	0.0022	1.21E-11
rs8049439	T	C	0.59	0.015	0.0023	6.99E-11
rs11774212	T	C	0.52	0.016	0.0023	1.51E-12
rs10483349	A	G	0.81	−0.017	0.0028	7.11E-10
rs71326918	A	C	0.1	0.022	0.0039	1.02E-08
rs11687170	T	C	0.83	0.021	0.0035	1.39E-09
rs7286601	T	G	0.54	−0.014	0.0023	1.99E-09
rs73344830	A	G	0.42	0.015	0.0023	9.93E-12
rs12143094	C	G	0.06	0.029	0.0049	2.73E-09
rs34638686	T	C	0.1	0.023	0.0038	1.51E-09
rs10761741	T	G	0.42	0.013	0.0023	7.05E-09
rs75090987	A	C	0.52	0.014	0.0022	1.14E-09
rs4500960	T	C	0.46	−0.014	0.0022	2.56E-10
rs1562242	T	C	0.48	−0.013	0.0022	5.95E-09
rs192818565	T	G	0.8	0.02	0.0029	2.02E-12
rs12534506	A	T	0.47	−0.014	0.0023	3.17E-10
rs10178115	T	G	0.54	0.014	0.0022	5.84E-10
rs62100765	T	C	0.42	−0.015	0.0023	1.08E-10
rs12142680	A	G	0.09	0.026	0.0043	8.97E-10
rs71413877	A	G	0.04	0.035	0.0058	1.91E-09
rs149613931	T	G	0.06	−0.028	0.0048	5.54E-09
rs17167170	A	G	0.8	0.019	0.0028	1.79E-12
rs12956009	T	C	0.57	−0.013	0.0022	3.75E-09
rs2179152	T	C	0.37	−0.013	0.0023	9.30E-09
rs7033137	C	G	0.76	0.015	0.0026	1.77E-08
rs4378243	T	G	0.83	0.018	0.0029	1.04E-09
rs4493682	C	G	0.17	0.019	0.003	1.54E-10
rs9755467	T	C	0.16	0.019	0.0031	5.11E-10
rs4851251	T	C	0.27	−0.015	0.0025	1.36E-09
rs7945718	A	G	0.62	0.014	0.0023	1.26E-09
rs1382358	T	C	0.87	0.02	0.0035	1.66E-08
rs148490894	A	G	0.98	0.044	0.0078	1.84E-08
rs12761761	T	C	0.24	0.016	0.0027	1.04E-08
rs142328051	T	C	0.91	0.022	0.0039	3.60E-08
rs55786114	T	C	0.07	−0.03	0.0045	4.11E-11
rs7948975	T	C	0.64	0.014	0.0023	1.14E-09
rs1606974	A	G	0.12	0.022	0.0034	1.82E-10
rs10772644	C	G	0.88	0.02	0.0035	1.65E-08
rs111321694	T	C	0.17	−0.016	0.003	4.33E-08
rs17425572	A	G	0.46	0.014	0.0022	1.38E-09
rs111730030	T	G	0.06	−0.029	0.005	7.51E-09
rs1550973	A	G	0.35	−0.014	0.0023	2.00E-09
rs2406253	A	G	0.81	0.016	0.0028	4.64E-08
rs7772172	A	G	0.4	0.013	0.0023	9.83E-09
rs281302	A	G	0.56	−0.013	0.0022	2.88E-09
rs17372140	A	G	0.3	−0.014	0.0024	9.19E-09
rs12640626	A	G	0.58	0.013	0.0023	1.66E-08
rs113011189	T	C	0.09	−0.025	0.0045	2.91E-08
rs56081191	A	G	0.07	0.028	0.0047	3.67E-09
rs12694681	T	G	0.69	0.014	0.0024	1.81E-08
rs12134151	C	G	0.5	−0.013	0.0022	1.14E-08
rs7914680	T	G	0.71	−0.014	0.0025	1.60E-08
rs6493271	T	C	0.83	0.017	0.0029	4.21E-09
rs152603	A	G	0.65	−0.013	0.0023	2.01E-08
rs7791133	A	C	0.38	−0.014	0.0023	2.33E-09
rs1389473	A	G	0.38	−0.013	0.0023	4.52E-09
rs61874768	T	G	0.18	−0.016	0.0029	3.85E-08
rs10818606	T	C	0.4	−0.014	0.0023	5.67E-10
rs2568955	T	C	0.25	−0.016	0.0026	5.77E-10
rs268134	A	G	0.25	0.014	0.0026	3.53E-08
rs6939294	T	C	0.23	0.016	0.0026	2.90E-09
rs12653396	A	T	0.56	−0.013	0.0022	7.65E-09
rs648163	T	C	0.26	0.014	0.0025	1.38E-08
rs140711597	C	G	0.98	0.052	0.0091	1.66E-08
rs301800	T	C	0.18	0.016	0.0029	2.85E-08
rs12462428	T	C	0.81	0.016	0.0028	3.31E-08
rs11756123	A	T	0.35	−0.015	0.0023	6.43E-11
rs7429990	A	C	0.27	−0.015	0.0026	8.44E-09
rs12702087	A	G	0.46	0.013	0.0022	1.74E-09
rs4076457	T	C	0.25	0.015	0.0026	8.85E-09
rs78387210	T	C	0.09	0.023	0.004	8.41E-09
rs7610856	A	C	0.43	0.012	0.0023	3.02E-08
rs78365243	T	C	0.95	0.029	0.0052	2.22E-08
rs1115240	C	G	0.75	−0.016	0.0026	7.05E-10
rs7605827	A	T	0.29	0.016	0.0029	4.86E-08
rs76076331	T	C	0.14	0.02	0.0032	2.38E-10
rs1596747	A	G	0.51	0.014	0.0022	1.14E-09
rs77702819	T	G	0.09	0.022	0.004	2.93E-08
rs12646808	T	C	0.66	0.015	0.0024	3.79E-10
rs2624818	A	G	0.11	0.021	0.0037	8.63E-09
rs7633857	C	G	0.52	−0.014	0.0026	4.74E-08
rs11976020	A	G	0.23	−0.015	0.0027	4.43E-08
rs4308415	C	G	0.44	−0.013	0.0022	2.52E-09
rs700590	T	C	0.59	−0.013	0.0023	2.84E-08
rs756912	T	C	0.52	−0.014	0.0022	1.14E-09
rs7241530	T	C	0.36	−0.013	0.0023	2.28E-08
rs35971989	A	G	0.84	0.018	0.0032	2.95E-08
rs11771168	T	C	0.24	−0.015	0.0027	2.56E-08
rs17504614	T	C	0.8	0.016	0.0028	1.56E-08
rs9914544	A	C	0.62	−0.013	0.0023	4.66E-08
rs4675248	A	G	0.4	−0.012	0.0023	4.39E-08
rs6800916	A	T	0.08	−0.024	0.0043	1.70E-08
rs35532491	A	T	0.9	-0.022	0.0038	7.15E-09
rs56044892	T	C	0.2	−0.016	0.0028	5.37E-09
rs79925071	T	G	0.56	0.013	0.0022	1.52E-08
rs12145291	T	C	0.94	−0.029	0.0051	2.21E-08
rs34106693	C	G	0.83	0.017	0.0031	1.80E-08
rs12754946	T	C	0.57	0.013	0.0023	1.48E-08
rs4741343	A	G	0.18	−0.016	0.0029	2.32E-08
rs76878669	C	G	0.76	0.014	0.0026	4.12E-08
rs775326	A	C	0.32	−0.014	0.0024	1.22E-08
rs10821136	T	C	0.34	0.013	0.0024	3.58E-08
rs1925576	A	G	0.54	−0.012	0.0022	2.23E-08
rs6065080	T	C	0.36	−0.013	0.0023	1.16E-08
rs56158183	A	G	0.07	0.025	0.0043	1.42E-08
rs12531458	A	C	0.52	0.012	0.0022	3.81E-08
rs62379838	T	C	0.69	0.013	0.0024	4.06E-08
rs7590368	T	C	0.73	−0.014	0.0025	2.72E-08
rs113520408	A	G	0.27	0.015	0.0025	7.15E-09
rs62263033	T	C	0.96	0.037	0.0063	5.60E-09
rs11643654	A	C	0.6	0.013	0.0023	2.00E-08
rs10930008	A	G	0.73	−0.014	0.0025	4.14E-08
rs56262138	A	T	0.3	0.014	0.0025	2.29E-08
rs113779084	A	G	0.31	0.014	0.0024	2.70E-08
rs62262721	T	C	0.96	0.042	0.0072	3.41E-09
rs1967109	A	G	0.15	−0.017	0.0031	4.40E-08

### IS GWAS Dataset

The IS GWAS dataset is from the METASTROKE consortium (Malik et al., 2016). The METASTROKE consortium performed a meta-analysis of 12 IS cohorts with a total of 10,307 IS individuals and 19,326 controls of European ancestry (*N* = 29,633 individuals) (Malik et al., 2016). More detailed information is described in the original study (Malik et al., 2016). The significance threshold for the association of these 162 educational attainment genetic variants with IS is *p* < 0.05/162 = 3.09 × 10^–4^.

### Pleiotropy Analysis

We performed a comprehensive pleiotropy analysis to assure that the selected genetic variants do not exert effects on IS through biological pathways independent of education levels. The American Heart Association and American Stroke Association have reported the leading risk factors for stroke, including high blood pressure, high cholesterol, heart disease (coronary artery disease), diabetes, current smoking, obesity, and excessive alcohol drinking (Meschia et al., 2014). In stage 1, we manually evaluated the potential pleiotropy using the GWAS datasets about the known confounders including high blood pressure, high cholesterol, body mass index (BMI), smoking behavior, and alcohol drinking from the UK Biobank (Sudlow et al., 2015); coronary artery disease from the CARDIoGRAMplusC4D [Coronary ARtery DIsease Genome wide Replication and Meta-analysis (CARDIoGRAM) plus The Coronary artery disease (C4D) Genetics] consortium (Nikpay et al., 2015); and type 2 diabetes from the DIAGRAM (DIAbetes Genetics Replication And Meta-analysis) consortium (Zhao et al., 2017). The significance threshold for the association of these 162 genetic variants with the potential confounders is a Bonferroni correction *p* < 0.05/162 = 3.09E-04.

In stage 2, we selected three statistical methods to perform the pleiotropy analysis. The first statistical method is based on the heterogeneity test (Greco et al., 2015; Hartwig et al., 2017; Liu et al., 2017). The potential heterogeneity in these genetic variants could be evaluated using Cochran’s Q test (together with the I^2^ index), which is a useful tool to explore the presence of heterogeneity due to pleiotropy or other causes, especially in MR studies with large sample sizes based on summary data (Greco et al., 2015). The second statistical method is the MR–Egger intercept test that provides an assessment of the validity of the instrumental variable assumptions and provides a statistical test of the presence of potential pleiotropy (Dale et al., 2017). The third statistical method is a newly developed method named Mendelian Randomization Pleiotropy RESidual Sum and Outlier (MR-PRESSO) test (Verbanck et al., 2018). In all these three statistical methods, the threshold of statistical significance for evidence of pleiotropy is *p* < 0.05.

### Mendelian Randomization Analysis

We selected three MR methods including inverse-variance-weighted meta-analysis (IVW), weighted median regression, and MR–Egger regression, as in recent studies (Dale et al., 2017; Larsson et al., 2017a; Tillmann et al., 2017; Liu et al., 2018; Liu et al., 2019). If there is no clear evidence of pleiotropy, these three methods should give consistent estimates. The odds ratio (OR) as well as 95% confidence interval (CI) of IS corresponds to a per 3.6 increase [about 1 standard deviation (SD)] in educational attainment levels. All analyses were conducted using R (version 3.2.4) and R package “MendelianRandomization” (Yavorska and Burgess, 2017). The statistical significance was *p* < 0.05.

### Power Analysis

The proportion of education variance explained by the instrumental variables can be estimated using *R* ^2^.

$$R^{2}=\sum_{i=1}^{k}\frac{\beta_{i}^{2}*2*MAF_{SNP_{i}}\left(1-\mathrm{MΑ}F_{SNP_{i}}\right)}{\mathrm{var}\left(X\right)}$$

where $\beta_{i}$ is the effect size (beta coefficient) associated with the education for $SNP_{i}$ , $MAF_{SNPi}$ is the minor allele frequency for $SNP_{i}$ , *K* is the number of genetic variants, and var(X) is the variance of the education [ var(X) = 1 for education, since the beta estimates refer to change in 1 standard deviation (SD)] (Pattaro et al., 2016; Mack et al., 2017). The strength of the instrumental variables was evaluated by the first-stage F-statistic (Noyce et al., 2017; Xu et al., 2017). A common threshold is F > 10 which avoids bias in MR studies (Burgess and Thompson, 2011). Here, we calculated statistical power to estimate the minimum detectable magnitudes of association for IS using the web-based tool mRnd and a two-sided type-I error rate *α* of 0.05 (Brion et al., 2013).

## Results

### Association of Educational Attainment Variants With IS

Of the 162 genetic variants associated with educational attainment, we extracted the summary statistics for all these 162 variants in the IS GWAS dataset. The characteristics of 162 genetic variants used as instrumental variables in IS are described in Supplementary Table S2. We noticed that none of these 162 genetic variants was significantly associated with IS risk at the Bonferroni-corrected significance threshold (*p* < 0.05/162 = 3.09 × 10^–3^) (Supplementary Table S2).

### Pleiotropy Analysis

In stage 1, 51 of these 162 educational attainment genetic variants are significantly associated with known confounders at the Bonferroni-corrected significance threshold (*p* < 0.05/162 = 3.09 × 10^–3^), as described in Supplementary Tables S3–S9. In brief, seven genetic variants were significantly associated with smoking. Two genetic variants were significantly associated with coronary artery disease. Six genetic variants were significantly associated with high blood pressure. 43 genetic variants were significantly associated with BMI. To meet the MR assumptions, we excluded these 51 genetic variants in the following analysis. In stage 2, using the remaining 111 genetic variants, the heterogeneity test showed no significant heterogeneity [I^2^ = 0%, 95% CI (0%; 16.8%), and *p =* 0.7093]. The MR–Egger intercept test showed no significant pleiotropy (MR–Egger intercept β = 0.018; *p* = 0.064). The MR-PRESSO test did not identify any horizontal pleiotropic outliers.

### Association of Educational Attainment Levels With IS

**FIGURE 1:**
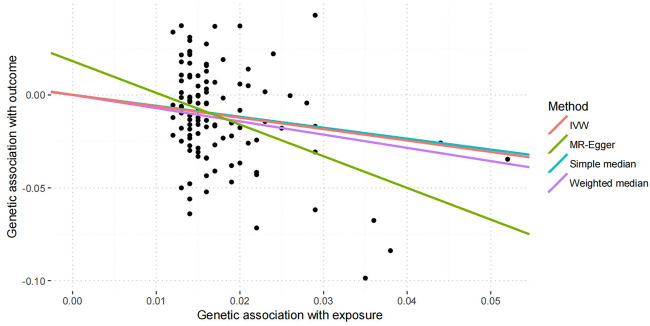
Individual causal estimates from each of the 111 genetic variants. This scatter plot shows individual causal estimates from each of 111 genetic variants associated with educational attainment on the *x*-axis and IS risk on the *y*-axis. The continuous line represents the causal estimate of educational attainment on IS risk.

Using the remaining 111 genetic variants, IVW showed that each SD increase in years of schooling (3.6 years) was significantly associated with a reduced IS risk (OR = 0.54, 95% CI: 0.41–0.71, and *p* = 1.16 × 10^–5^). Interestingly, the estimates from weighted median (OR = 0.49, 95% CI: 0.33–0.73, and *p* = 1.00 × 10^–3^), and MR–Egger estimate (OR = 0.18, 95% CI: 0.06–0.60, and *p* = 5.00 × 10^–3^), were consistent with the IVW estimate in terms of direction and magnitude, as provided in Table 2. Figure 1 shows individual causal estimates from each of the 111 genetic variants using different methods.

**TABLE 2:** MR analysis results between educational attainment and IS.

Method	OR	95% CI	*p* value
Inverse-variance weighted	0.54	0.41–0.71	1.16 × 10^–5^
Weighted median	0.49	0.33–0.73	1.00 × 10^–3^
MR–Egger	0.18	0.06–0.60	5.00 × 10^–3^

OR, odds ratio; CI, confidence interval; the significance was at *p* < 0.05.

### Power Analysis

Here, all these 111 genetic variants could explain about 1.09% of the educational attainment variance (*R* ^2^ = 1.09%). The first-stage F-statistic for the instrument including these 111 genetic variants was 327.56 > 10, so a weak instrument bias is unlikely. The actual N for IS GWAS is 29,633, and the proportion of cases is 0.347822. Our MR study had 80% power to detect effect sizes of moderate magnitude with ORs as low as 0.71 and as high as 1.37 per SD increase in educational attainment levels for IS. Importantly, the power to detect the causal association (OR = 0.54, 95% CI: 0.41–0.71, and *p* = 1.16 × 10^–5^) is 100% by selecting these 111 genetic variants as the instrumental variables. Hence, our analysis has enough statistical power to detect robust causal effect estimates.

## Discussion

It has been a long time since the relation between the educational attainment and risk of stroke was evaluated. Until November 2015, there have been 79 observational studies including approximately 164,683 strokes (Mchutchison et al., 2017). However, these observational studies have reported both positive and negative associations between higher educational attainment and stroke (Mchutchison et al., 2017). Meanwhile, there was clear between-study heterogeneity in all comparisons, ranging from 76% to 96% (Mchutchison et al., 2017). Until now, it has been difficult to establish causality because of methodological limitations of traditional observational studies.

Here, we performed an MR analysis to evaluate the potential association of educational attainment with IS risk using large-scale GWAS datasets. MR is based on the premise that the human genetic variants are randomly distributed in the population (Emdin et al., 2017). These genetic variants are largely not associated with confounders and can be used as instrumental variables to estimate the causal association of an exposure with an outcome (Emdin et al., 2017), which could avoid the methodological limitations of the traditional observational studies.

Our results indicated that a genetically increased educational attainment was significantly associated with reduced IS risk. IVW showed that each additional 3.6-year increase in years of schooling was significantly associated with a reduced IS risk (OR = 0.54, 95% CI: 0.41–0.71, and *p* = 1.16 × 10^–5^). Importantly, other sensitivity analyses further supported this estimate. All these findings show that the causal association between genetically increased educational attainment and reduced IS risk is robust. Hence, our results do seem to hint at what lifestyle choices may help protect against IS. The life experiences that engage the brain, such as higher educational attainment, may protect against IS risk.

Our findings are comparable to findings from traditional observational studies with OR = 0.74 (Mchutchison et al., 2017), 0.65 (men) (Ferrario et al., 2017), and 0.71 (women) (Ferrario et al., 2017). Meanwhile, our findings are also consistent with the results from a recent MR study, which found that one SD increase in years of schooling (3.6 years) was associated with a reduced risk of coronary heart disease (OR = 0.67, 95% CI 0.59–0.77; *p* = 3.00 × 10^–8^) (Tillmann et al., 2017). It has been established that coronary artery disease is one of the leading risk factors for stroke (Meschia et al., 2014).

Until now, 3 MR studies have also investigated the causal association between educational attainment and IS. Harshfield et al. assessed the causal effect of 12 lifestyle factors on risk of stroke (Harshfield et al., 2021). They found that genetically increased educational attainment was associated with reduced risk of IS, large artery stroke, and small vessel stroke, and intracerebral hemorrhage using 305 educational attainment genetic variants (Harshfield et al., 2021). Wen et al. selected 58 educational attainment genetic variants and identified a suggestive causal association between education and IS (*p* = 0.048) (Xiuyun et al., 2020). Gill et al. selected 625 instrument SNPs for educational attainment and found that education was causally associated with stroke risk (Gill et al., 2019). A main difference between our and previous MR studies is the manual pleiotropy analysis. These above 3 MR studies only used the statistical methods to perform the pleiotropy analysis (Gill et al., 2019; Xiuyun et al., 2020; Harshfield et al., 2021).

This MR study has several strengths. First, this study may benefit from the large-scale educational attainment GWAS dataset (*N* = 405,072 individuals of European descent individuals) and IS GWAS dataset (*N* = 29,633 individuals of European descent). Importantly, power analysis further provides ample power to detect the association of educational attainment with IS risk. Second, both the educational attainment and IS GWAS datasets are from the European descent, which may reduce the influence on the potential association caused by the population stratification. Third, multiple independent genetic variants are taken as instruments, which may reduce the influence on the potential association caused by the linkage disequilibrium; Fourth, we selected multiple methods to perform MR analysis, as in previous studies (Mokry et al., 2015; Nelson et al., 2015; Emdin et al., 2017; Larsson et al., 2017a; Manousaki et al., 2017; Noyce et al., 2017). Fifth, we performed a comprehensive pleiotropy analysis to evaluate the potential association of these educational attainment genetic variants with known IS risk factors. We excluded 51 genetic variants associated with potential confounders, which meets the MR assumptions.

Despite these interesting results, we recognize some limitations in our study. First, we could not completely rule out that there may be additional confounders, although some other available software or tools may be helpful to identify the pleiotropy, such as GSMR (Zhu et al., 2018) and CAUSE (Morrison et al., 2020). Until now, it is almost impossible to fully rule out pleiotropy present in any MR study (Emdin et al., 2017; Larsson et al., 2017a; Larsson et al., 2017b). Second, it could not be completely ruled out that population stratification may have had some influence on the estimate. Third, the genetic association between education and IS may be different in different ancestries. Hence, this causal association should be further evaluated in other ancestries. In some individuals, the association between a genetic variant and one specific outcome may have been confounded by the hidden population structure (Davies et al., 2018). Thus, MR studies using these individuals could have been biased by population stratification or different ancestries (Davies et al., 2019). In fact, Zheng et al. found that hypertension could play different causal roles on chronic kidney disease across ancestries (Zheng et al., 2022). Fourth, the underlying mechanisms about the causal association between educational attainment and IS remain unclear.

In summary, we provide genetic evidence that high education could reduce IS risk. Our findings could have public health implication to raise awareness of the extent to which educational inequalities are associated with risk of IS. Meanwhile, population-based solutions may contribute to ameliorate the deleterious effects of low educational attainment on health outcomes.

## Data Availability

The original contributions presented in the study are included in the article/Supplementary Material, further inquiries can be directed to the corresponding authors.

## Abbreviations

BMI, body mass index; CI, confidence interval; CARDIoGRAMplusC4D, Coronary ARtery DIsease Genome wide Replication and Meta-analysis (CARDIoGRAM) plus The Coronary Artery Disease (C4D) Genetics) consortium; DIAGRAM, DIAbetes Genetics Replication And Meta-analysis consortium; IS, ischemic stroke; GWAS, genome-wide association study; IVW, inverse-variance-weighted meta-analysis; OR, odds ratio; SD, standard deviation; SNP, single-nucleotide polymorphism; SSGAC, Social Science Genetic Association Consortium.
